# VACCINATION MOTIVATORS AND DETERRENTS AMONG UNDERVACCINATED OLDER ADULTS IN NORTH DAKOTA

**DOI:** 10.1093/geroni/igae098.2476

**Published:** 2024-12-31

**Authors:** Andrea Huseth-Zosel, Heather Fuller, Paul Carson

**Affiliations:** North Dakota State University, Fargo, North Dakota, United States; North Dakota State University, Fargo, North Dakota, United States; North Dakota State University, Fargo, North Dakota, United States

## Abstract

Despite increased risk of morbidity and mortality among older adults due to preventable infectious diseases such as influenza, shingles, pneumonia, and COVID-19, many older individuals forego receiving some, if not all, of these vaccinations. This study examines vaccination motivators and deterrents for undervaccinated older adults in North Dakota (ND). Adults aged 65+ in ND were mailed a survey (n=901) with questions gauging vaccination behaviors and perceptions. Of these participants, 132 indicating not receiving influenza, shingles, pneumonia, or COVID-19 vaccinations. Further questions assessed reasons they had not been vaccinated against each specific disease (e.g., “Concerned about side effects”, “Vaccines are dangerous”, “I’m healthy and I do not need it”) and what would make it more likely to get a vaccine (e.g., “More information”, “Doctor recommendation”, “Easy access to vaccines”). Reasons for remaining unvaccinated varied by vaccine type. For influenza and pneumococcal vaccines, respondents were more likely to indicate they are healthy and do not need the vaccine. For shingles and COVID-19, respondents were more likely to indicate concerns about side effects. Factors reported to motivate increasing the likelihood of getting any vaccine were receiving a doctor recommendation, receiving more information, and having a vaccine provided at no cost. These results contribute to our understanding of vaccination behaviors among older adults and underscore specific issues around which to frame interventions tailored to increase vaccine uptake for this population.

